# CUDAICA: GPU Optimization of Infomax-ICA EEG Analysis

**DOI:** 10.1155/2012/206972

**Published:** 2012-07-03

**Authors:** Federico Raimondo, Juan E. Kamienkowski, Mariano Sigman, Diego Fernandez Slezak

**Affiliations:** ^1^Departamento de Computación, Pabellón I, Ciudad Universitaria, C1428EGA Ciudad Autonoma de Buenos Aires, Argentina; ^2^Laboratory of Integrative Neuroscience, Physics Department, University of Buenos Aires, Buenos Aires, Argentina

## Abstract

In recent years, Independent Component Analysis (ICA) has become a standard to identify relevant dimensions of the data in neuroscience. ICA is a very reliable method to analyze data but it is, computationally, very costly. The use of ICA for online analysis of the data, used in brain computing interfaces, results are almost completely prohibitive. We show an increase with almost no cost (a rapid video card) of speed of ICA by about 25 fold. The EEG data, which is a repetition of many independent signals in multiple channels, is very suitable for processing using the vector processors included in the graphical units. We profiled the implementation of this algorithm and detected two main types of operations responsible of the processing bottleneck and taking almost 80% of computing time: vector-matrix and matrix-matrix multiplications. By replacing function calls to basic linear algebra functions to the standard CUBLAS routines provided by GPU manufacturers, it does not increase performance due to CUDA kernel launch overhead. Instead, we developed a GPU-based solution that, comparing with the original BLAS and CUBLAS versions, obtains a 25x increase of performance for the ICA calculation.

## 1. Introduction

Analysis of brain imaging data has two intrinsic difficulties: dealing with high volumes of data (and often high dimensional) and a usually low signal-to-noise ratio due to persistent artifacts. A significant number of methods have been developed, usually based on some form of dimensionality reduction of data, to cope with these difficulties. Multivariate statistical analysis for the separation of signals is a widely studied topic of great complexity because of the large number of sources and the low signal-to-noise ratio, inherent in this kind of signals. Specific approaches have been developed to separate the signals generated by the study of those sources that contribute only noise, such as principal component analysis (PCA) [1], factor analysis [2], and projection pursuit [3], among others.

Independent Component Analysis (ICA) [4–6] is one of the most effective methods for source separation and removal of noise and artifacts. The most emblematic example was the separation of audio sources in noisy environments [5]. In recent years, it has become a standard in brain imaging-electroencephalogram (EEG) [7–9], magnetoencephalogram (MEG) [10] and functional magnetic resonance imaging (fMRI) [11–13]. It has been used for the removal of artifacts arising from eye movements [14], but also for the selection of relevant dimensions of the data [15, 16]. In fact, the most popular open packages for EEG analysis—EEGLAB (http://sccn.ucsd.edu/eeglab/) and Fieldtrip (http://fieldtrip.fcdonders.nl/)—strongly rely on ICA.

Analyzing EEG, MEG, or fMRI with ICA does not come without a cost and requires a huge amount of computing power. For example, analyzing a typical single-subject EEG experiment (data from 132 channels, at 512 Hz of sampling rate, stored in single-precision, for a 1-hour experiment, which amounts to a total of 1.5 GB of data), typically takes around 12 hours on an Intel i5, 4 GB RAM, or 8.5 hours on an Intel i7, 16 GB RAM. Often one needs to look at the output of ICA before making a decision on how to proceed with the data, and this long-lasting processing heavily conditions the flexibility of analysis. More importantly, it makes this analysis completely prohibitive for online access of the data, for instance in Brain Computer Interface applications.

Current standard CPU hardware include different extensions of the basic instruction set architecture for vector processing support, for example, the Streaming SIMD Extensions (SSE) or Advanced Vector Extensions (AVX). These extensions enable the parallel execution of the same operations on multiple data, a key requirement for signal-processing. Although, no effective tools to operate with more than a few tens of floating point numbers simultaneously are available in current CPUs, which makes the calculation more efficient but it still lacks the key feature needed to get the results quick. In other words, processors of coming years will only improve slightly this performance.

One approach to solving this problem is using Beowulf parallel computing clusters [17]. The main drawback of this implementation is the communication overhead needed to synchronize the different compute nodes, since the memory is distributed over the nodes.

Here, we propose to use of the massive parallel processors included in the graphical units (GPU) for ICA calculations. Contrary to a Beowulf, this architecture has a common shared memory allowing a much faster parallelization of ICA algorithms. In addition, the low cost of GPUs makes this project available for virtually every user. We investigate the processing of EEG data series using CUDA: a parallel computing platform and programming model. CUDA extends C/C++ programming language, enabling the programmer to write a serial program (functions, also called *cuda kernels*) that executes in parallel across a set of threads operating over different memory positions [18].

Many implementations of ICA are available, for example, Infomax [5], SOBI [19], and FastICA [20]. These implementations use mostly linear algebra operations which are included in off-the-shelf optimized GPU standard libraries. FastICA has demonstrated that parallelization may be relatively straightforward replacing linear algebra routines by standard GPU libraries [21].

However, ICA of human EEG data is much better approximated by Infomax [7] enhanced which has made it a standard in EEG analysis. We show that simply replacing linear algebra routines with GPU libraries do not show better performance. Parallelizing Infomax requires an efficient optimization of GPU memory access and kernel dispatch, in order to obtain an increased performance. Here, we set to develop and implement CUDAICA, an optimization algorithm to increase processing time by a 25x factor, at almost no cost, without changing the original algorithm.

## 2. Independent Component Analysis

ICA was introduced in 1994 by Comon [22] independent. The concept of ICA can be seen as an extension of the PCA, where the linear transformation minimizes the statistic dependence between its components.

The following statistic model is assumed [23]:

(1)x=My+v,

where *x*, *y*, and *v* are random vectors with values in ℝ or *ℂ* with zero mean and finite covariance, *M* is a rectangular matrix with at most as many columns as rows and vector *y* has statistically independent components.

The problem set by ICA can be summarized as follows: given *T* samples of vector *x*, an estimation of matrix *M* is desired, and the corresponding samples from vector *y*. However, because of the presence of noise *v*, it is in general impossible to reconstruct the exact vector *y*. Since the noise *v* is assumed here to have an unknown distribution, it can only be treated as a nuisance, and the ICA cannot be devised for the noisy model above. Instead, it will be assumed that:

(2)x=As,

where *s* is a random vector whose components are maximizing statistical independence [22].

Both, EEGLAB and FieldTrip analysis software, use the Infomax algorithm [5] for estimation of independent components [24, 25]. Infomax is based on a neural network with three columns of neurons, each representing: (1) the original data (*X*); (2) the registered data (*r*); (3) the approximated independent data (*Y*). Each column of neurons combine linearly by matrices *A* and *W*.

The principle used by this algorithm is maximizing the mutual information that output *Y* of a neural network processor contains about its input *X*, defined as

(3)I(Y,X)=H(Y)−H(Y ∣ X),

where *H*(*Y*) is the entropy of output *Y* and *H*(*Y* | *X*) is the entropy of the output that did not come from the input. In fact, *H*(*Y*) is the differential entropy of *Y* with respect to some reference, such as the noise level or the accuracy of discretization of the variables in *X* and *Y*. Thus, only the gradient of information-theoretic quantities with respect to some parameter *w* is considered [5]. Then, the equation (3) can be differentiated, with respect to a parameter *w* as:

(4)∂∂wI(X,Y)=∂∂wH(Y),

because *H*(*X* | *Y*) does not depend on *w*.

In the system (1), *H*(*X* | *Y*) = *v*. Whatever the level of the additive noise, maximization of the mutual information is equivalent to the maximization of the output entropy, because (∂/∂*w*)*H*(*v*) = 0. In consequence, for any invertible continuous deterministic mappings, the mutual information between inputs and outputs can be maximized by maximizing the entropy of the outputs alone.

The natural (or relative) gradient method simplifies considerably the method. The natural gradient principle [26, 27] is based on the geometric structure of parameters space and it is related to the relative gradient principle [28] that ICA uses.

Using this approach, the authors propose the following iteration of the gradient method to estimate the *W* matrix:

(5)ΔW∝W−tanh(Wx2)(Wx)TW.

In summary, Infomax ICA consists of the following steps:

1. *U* = *W* × perm(*x*) (where perm is a random permutation),
2. *Y* = −tanh(*U*/2),
3. *YU* = *Y* × *U* ^*T*^,
4. *YU* = *YU* + *I*,
5. *W* = *l*rate × *YU* × *W* + *W*,

where *l*rate is the learning rate for each iteration of the method, generally lower than 1*e* ^−2^. High values of *l*rate may lead to faster computation but a bad choice could destroy convergence. We propose a fast implementation using GPUs keeping the original algorithm intact, including the selection criteria for *l*rate.

## 3. Material and Methods

We first present, as a baseline measure, the performance of Infomax ICA implementations available. We then compare them with our development for Infomax ICA based on CUDA optimized for GPU processing.

### 3.1. Infomax ICA Implementations

Current implementations (C/C++ and Matlab) make use of Basic Linear Algebra Subprograms (BLAS). This library provides the standard routines for performing basic vector and matrix operations [29]. Several implementations of BLAS can be used to compute ICA: ATLAS, Intel MKL, and CUBLAS, among others.

The most popular implementation of these routines is ATLAS, a portable self-optimizing BLAS, included in most unix distributions [30]. Intel offers the Math Kernel Library (Intel MKL), a computing math library of highly optimized, extensively threaded math routines. NVIDIA offers CUBLAS, an implementation of BLAS on top of the NVIDIA CUDA driver for GPU optimization of linear algebra routines.

Generally, EEG equipment represents electrode data as a time series of single-precision floats. Nevertheless, all implementations of Infomax ICA use double-precision floats to ensure numerical stabilities and avoid numerical error propagation due to precision. Thus, a GPU with CUDA compute capability 1.3 (or greater) must be used, in order to support double-precision float operations.

### 3.2. Testing Hardware

Performance tests of ICA computation have been made on several hardware configurations. Three different CPUs where used: i7-2600, i5 430M, and Xeon E541a. For GPU technology, we used a Tesla C2070 and GTX 560. To compare the performance of our solution, we chose the high-end equipment: the Intel Core i7-2600 with 16 GB of RAM and the Nvidia Tesla C2070 video card. Some tests have been done with an Nvidia Quadro 4000 which showed a comparable performance to Tesla hardware.

Performance comparisons were made using real datasets with the number of channels varying from 32 to 256 in 32 channel steps. The amount of samples vary from 15 minutes experiments to 105 minutes in 15 minutes steps, with a sampling rate of 512 Hz.

### 3.3. Testing Datasets

The testing experiment consists of 70 trials where the subject freely explores between a set of dots distributed along the horizontal line. The trial starts with the participant fixating in a small dot in the bottom half of the screen, and the set of dots appear in the top half of the screen. Participants have 5 seconds to explore and typically perform 15 saccades in each trial. Participants are instructed to explore in random order and are free to blink. We purposely gave this instruction to have data contaminated by typical eye-movement and blinking artifacts. Simultaneous eye movement and EEG were recorded using eye tracker Eyelink 2K, SR-Research, and Active-Two EEG with 128-channel, Biosemi system.

## 4. Results

### 4.1. Infomax ICA Profiling

We performed a detailed analysis of all the operations involved in the execution of ICA using *callgrind*, a tool for sequential profiling and optimization [31]. We executed the method for different datasets and profiled the function calls, observing that BLAS routines dgemv and dgemm consumed more than 80% of total calculation time. For instance, dgemv and dgemm take, respectively, 47.9% and 40.78% of the total amount of time used to calculate ICA for a 136 channels dataset and 22528 samples, recorded at 512 Hz. Thus, we selected these as the most relevant candidates for GPU optimization. The dgemv and dgemm symbols correspond to BLAS functions for matrix-vector and matrix-matrix multiplication, respectively.

### 4.2. MKL Implementation

The easiest way to achieve significant performance increment in applications with substantial amounts of BLAS operations is using the available optimized libraries developed by the processor manufacturers, the Intel MKL Libraries. These libraries use multithreaded implementations of BLAS operations and exploit all features of installed processors. This upgrade is extremely simple since no code or compiler commands are needed to be modified. Instead, simply compiling and linking to MKL libraries provides a more efficient execution of Infomax ICA with multithreaded BLAS operations.

Using this multithreaded version, we compared the performance of MKL Infomax ICA versus the standard one, on a Intel Core i7. ATLAS implementation takes 1.5 seconds per step on the smallest experiment (32 channels, 15 minutes of experiment) and grows almost linearly as channels and total time increase, reaching 620 seconds per step on the biggest experiment (256 channels, 105 minutes of experiment), as shown in Figure 3(a). MKL implementation shows a significant increase of performance, showing a maximum of 142 seconds per step of experiment (see Figure 3(a)). We obtained a maximum speedup of 4.5 using the MKL libraries for the experiments with more channels (see Figure 3(b)).

### 4.3. CUDAICA Implementation

The time series nature of data, make GPU computing a very promising approach for EEG processing optimization. A naive and simple optimization can be done using CUBLAS—NVIDIA implementation of BLAS on top of the NVIDIA CUDA driver—replacing symbols dgemv and dgemm with CUBLAS symbols cublasDgemv and cublasDgemm, respectively. This approach was first used for a basic optimization of FastICA [21].

#### 4.3.1. CUBLAS Approach

CUBLAS uses a different memory space than standard sequential calculations, that is, the video card RAM. Thus, before starting computation, data must be transferred from CPU memory to video memory. This results in a few additional calls to memory movement operations.

Performance measurements revealed that replacing the BLAS routines to their corresponding CUBLAS routines did not increase processing speed. The amount of time required to process the experiment was longer than ATLAS. In 32 and 64 channels, CUBLAS-implementation took 6.5x and 2.4x times longer than the ATLAS implementations. Only with 128 channels, the CUBLAS-implementation performance equals to ATLAS implementation, suggesting that if no effort wants to be invested in an ad-hoc solution, MKL is the best solution.

This lack of improvement is caused by the constant overhead of running the parallel kernels on GPU. Before starting each iteration, Infomax ICA creates a vector with a random permutation of the indexes, from 0 to *N* − 1, being *N* the number of samples in the dataset. Then, matrix *W* is multiplied by the column of *x* as indicated by the index in the random permutation vector using the corresponding BLAS matrix-vector operation. This results in as many matrix-vector multiplication as the numbers of samples in the dataset instead of a small number of matrix-matrix multiplications. The execution of each iteration results in an accumulated overhead that is only compensated with high dimensional datasets, where each step involves many operations.

As mentioned before, we observed matrix-vector and matrix-matrix multiplications consumes about 50% and 40% percent of the total amount of time required to compute Infomax, respectively. We performed performance tests comparing ATLAS against CUBLAS routines on matrix-matrix and matrix-vector operations using scenarios from Infomax algorithm. The results were conclusive: matrix-matrix operations were faster using CUBLAS, but matrix-vector operations were the bottleneck in this computation. Due to the actual dimensions of matrix *W* and the size of the column of *x*, ATLAS performed faster. The performance increase achieved with CUBLAS for the matrix-matrix operations (40% of total time) was opaqued by the overhead involved in execution of GPU functions and the lack of performance gain in the matrix-vector computation.

#### 4.3.2. Hybrid Approach: CUDAICA

**Figure 1:**
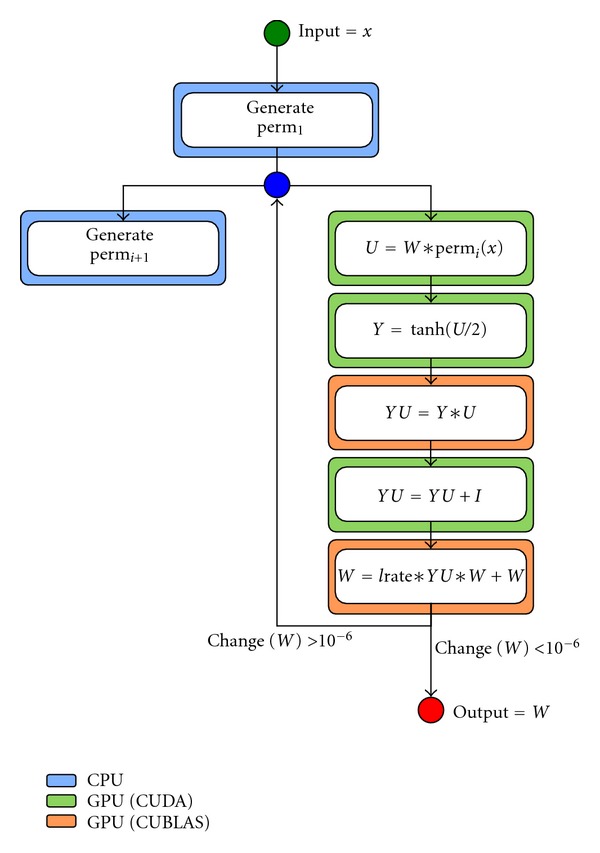
Flow diagram of hybrid implementation. We divide operations in three groups: CPU (blue box), GPU using standard CUBLAS libraries (orange box), and GPU using own CUDA implementation (green box).

Based on this profiling, we implemented an hybrid solution of the method using CUDA (CUDAICA). The main objective of this implementation was to keep the original algorithm intact: reduce the computation time of the same operations. All the matrix-matrix operations are computed using CUBLAS and the remaining operations are solved by an ad-hoc implementation based on CUDA (see Figure 1).

Initially, all matrix and vectors are copied into the GPU device global memory. In the process, single precision floats—from the EEG time series—are converted to double precision. The optimizations applied to Infomax ICA consisted on two main optimizations: (1) optimizations on memory access and kernels executions in particular matrix operations (*U* = *W*∗perm(*x*), *Y* = −tanh(*U*/2), and *YU* = *YU* + *I*) and (2) combining asynchronous execution in CPU and GPU to generate each step permutation perm_*i*+1_ of vector *x*, while GPU is computing step *i*.

Taking advantage of CUDA shared memory space, this optimization first copies the vector indexed by the permutation into shared memory. Once transferred, *U* is computed by an ad-hoc matrix-vector multiplication implementation. In the same kernel, as matrix *U* is being calculated, matrix *Y* is computed by computing −tanh(*z*/2), where *z* represents the values being computed in *U*. This combined operations reduce significantly the number of kernels launched and therefore the overhead involved in kernel initialization.

The amount of shared memory depends on GPU compute capability version. Since version 2.0, CUDA supports double-precision floating points operations and 48 KB of shared memory. These features allow to compute matrix-vector operations for dimensions up to 6144 elements at a time. By optimizing the algorithm implementation to use aligned memory, we obtained optimum memory access resulting in 128 Byte transactions to main memory without discarding any data.

Other significant improvement is made when computing *YU* = *YU* + *I*: instead of using the generic sum function, a specific CUDA kernel is used to modify only the elements in the diagonal of *YU*.

Random permutations can be performed by generating an index vector of random indexes in CPU while GPU computation is being performed. Then, when permutation is needed the random vector is used to index in the data matrix to locate the corresponding randomly permuted vector.

**Figure 2:**
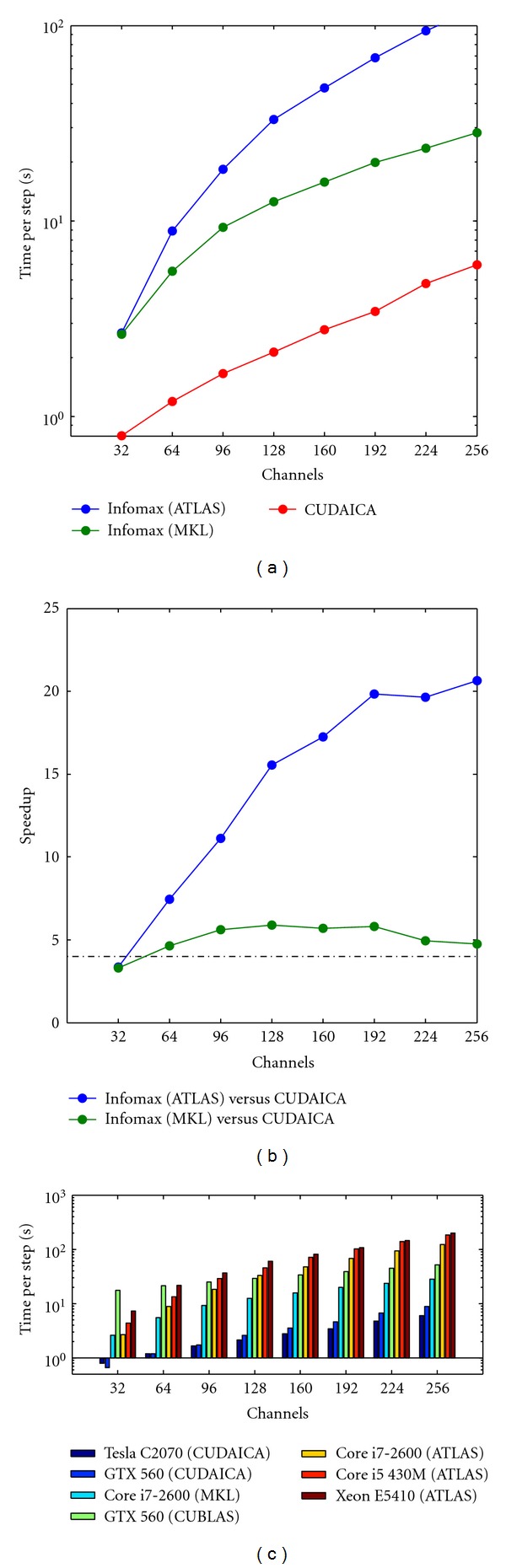
Execution time of Infomax step using ATLAS, MKL, and CUDAICA for 30-minute experiments (a). This short experiments may last more than 4 hours of computation time in the standard BLAS implementation with 256 channels. MKL shows significant performance improvement over BLAS, and CUDAICA behaves the best. Comparison between ATLAS and MKL versus CUDAICA (b). CUDAICA shows a maximum speedup of 4.7 over MKL, and more than 20x over standard ATLAS implementation. In (c), we show the time per step of different implementations of Infomax with several channel configurations, running under the available hardware.

Combining these tweaks, we obtained an optimized version of Infomax ICA, with a processing time of less than 0.5 seconds per step in the 30-minute experiment with 32 channels (see Figure 2(a)).

In Figure 2(a) we show the performance comparison in a 30-minute experiment between CUDAICA and both Infomax implementations: ATLAS and MKL based. We observe an exponential growth of time per step (note de log scale in *y*-axis) in the MKL and CUDAICA implementations. ATLAS show a decrease of slope as channels increase, but always bigger than MKL and CUDAICA. Starting from 96 channels, MKL and CUDAICA show a similar slope, indicating an almost constant increase of performance of CUDAICA over MKL of 4.5x (Figure 2(b)). We obtained a maximum performance increase of CUDAICA versus the ATLAS implementation of more than 20x, for the experiments of 192 channels or more. Interestingly, we found that CUDAICA performed best versus MKL for 128 channels, the most typical configuration of our EEG setup.

All performance comparisons were performed in the best available hardware: i7 and Tesla C2070. In Figure 2(c), we show the time per step of the method in the different hardware available, with several channel configurations. As expected, we observe an almost linear increase of all runs as number of channels grow. Comparison between standard CPU and GPU processing shows a significant performance increase using any GPU (top-of-line Tesla or standard GTX560 card) against the non-GPU hardware tested.

### 4.4. Reliability of CUDAICA Relative to Previous Algorithms

The new optimization involves reordering of operations which may potentially affect the numerical stability of the calculations. Thus, we verified that CUDAICA produces results which are not distinguishable from previous implementations. For this purpose, we removed the random vector generation used in the first part of the method. After each step, we compared the output for the same inputs and the exact same result was achieved.

**Figure 3:**
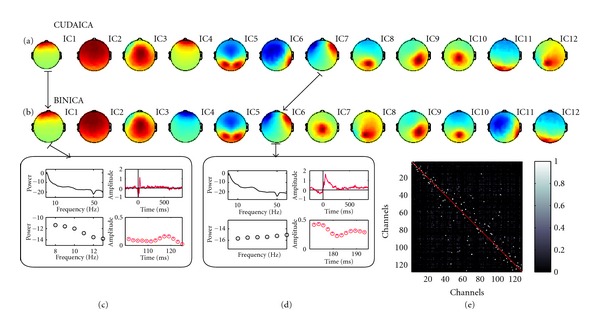
Comparison between CUDAICA and original code estimated components (ICs): (a, b) first twelve ICs of CUDAICA corresponds to the first twelve ICs, note that the order could be switched and the weights could be inverted (IC4). (c, d) Example of two pairs of components, IC1-IC1 and IC7-IC6, respectively. Both spectra (top-left panels) and eye-movement artifact locked to saccade offset (top-right panels) are indistinguishable by naked eye. We also show an amplified regions where it is possible to appreciate that there are two curves in each plot (bottom panels). Black and red lines: CUDAICA, and grey and magenta lines: Infomax ICA. (e) Average trial-by-trial correlation between pairs of ICs. Almost all rows have a single white spot corresponding to the matched IC calculated by the other implementation, and these spots are not necessarily aligned in the diagonal. Note that there are some subsets of components that do not have the correspondent ICs as these subsets correspond to a subset in other implementation.

Then, we compared the output of the full algorithm using the original code (ATLAS based) and CUDAICA, executing both implementations and estimating the independent components of the same dataset. In Figure 3 we show the first 12 independent components calculated with both implementations (Figures 3(a) and 3(b)). As expected, executions show similar (but not identical) independent components, due to its random nature—that is, some ICs were in different order or with inverted weights. The first 3 components show almost the exact same behavior. As an example, in Figures 3(c) and 3(d) we plot the spectra and eye-movement artifact locked to saccade offset for IC1 and IC7-IC6 of both implementations. Difference are indistinguishable (correlation coefficient *R* = 1, *p* < 0.0001); we also show an amplified region where is possible to appreciate that there are, indeed, two curves in each plot in magenta (original code) and red (CUDAICA). We calculate the average trial-by-trial correlation between pairs of ICs of ATLAS and CUDAICA runs (Figure 3(e)). Almost all rows have a single white spot corresponding to the matched IC calculated by the other implementation. This spots are not necessarily aligned in the diagonal, as order could be switched and even weights could be inverted.

## 5. Discussion and Conclusions

ICA is one of the de-facto standard methods for source separation and removal of noise and artifacts. In neuroscience, it has been widely used for EEG [7, 8], fMRI [12, 14], and invasive electrophysiology [32].

In all these neuroimaging methods, technology has increased the data volume, improving spatial and temporal resolution. With current standards, analyzing data with ICA requires a vast, often intractable amount of computing power.

In practical EEG analysis, this computer power requirements impedes the rapid exploration of different methods since each implementation of ICA runs overnight or even taking more than one day. A rapid iteration and examination of different procedures becomes completely impractical. Even more, ICA is difficult to use for online access of the data. Over the last years there has been an exponential development of brain computing interfaces which require online access to the relevant dimensions of the data [13, 33–35].

ICA constitutes a formidable tool for finding relevant directions and BCI procedures [36] which use ICA to present participants with different components to determine which are easier to control is a timely necessity. For this, it is imperative to implement ICA at much faster speeds than is being implemented with current CPU and here we present a major advancement in this direction.

Our aim here was purely methodological: improve the speed of Infomax ICA by at least 10x. We performed a detailed profiling and detected the bottleneck in the calculation of independent components, showing that vector-matrix and matrix-matrix operations take almost all computational time. Based on these results, we implemented an hybrid ad-hoc solution for GPU optimizations: CUDAICA. With this solution, we compared CUDAICA to the original BLAS (compiled with standard ATLAS and the optimized MKL libraries) and CUBLAS implementations. We observed a 25x performance increment using CUDAICA, over the standard ATLAS implementation, and 4.5x performance increment compared to the MKL implementation.

With this calculation time, a 128-electrodes EEG of 1-hour experiment would take 1500 seconds approximately to compute the independent components. This timing opens up new possibilities of the method, for instance for Brain Computer Interface applications, making possible to think of an experiment where Independent components may be calculated during the experiment and use them as a feedback feature.

CUDAICA was developed under the GNU General Public License, and is freely available from our wiki with a description of application features, FAQ and installation instructions (http://calamaro.exp.dc.uba.ar/cudaica/doku.php?id=start). CUDAICA woks as a standalone application and integrates to the EEGLAB Toolbox adding an option to process ICA using CUDAICA, just like any other ICA implementation. It was designed for standard EEGLAB users, with no extra effort needed to run this implementation. It works under CUDA enabled hardware, that is, almost every modern graphic card, making CUDAICA widely available and easy to use.
